# Renal proteomics of male offspring exposed to maternal protein restriction: molecular, epigenetic, and nephron-specific signatures of metabolic programming

**DOI:** 10.1007/s13105-026-01189-9

**Published:** 2026-05-07

**Authors:** Danielle Amanda Niz Alvarez, Isabelle Tenori Ribeiro, Matheus Naia Fioretto, Marina Pereira Pires, Luisa Annibal Barata, Flávia Alessandra Maciel, Luiz Marcos Frediane Portela, Renato Mattos, Hecttor Sebastian Baptista, Pedro Menchini Vitali¹, Vinicius Alexandre Andrade Felipe, Marcel Rodrigues Ferreira, Elena Zambrano, Patrícia Aline Boer, Luis Antonio Justulin

**Affiliations:** 1 Department of Cellular and Molecular Biology, Institute of Biosciences, Sao Paulo State University, Botucatu, SP Brazil; 2 Molecular Genetics and Bioinformatics Laboratory, Experimental Research Unit - Unipex, School of Medicine, São Paulo State University - Unesp, Botucatu, São Paulo Brazil; 3 Departamento de Biología de la Reproducción, Instituto Nacional de Ciencias Médicas y Nutrición Salvador Zubirán, Mexico City, México; 4 Departamento de Biología - Facultad de Química, Universidad Nacional Autónoma de México, Mexico City, México; 5 Fetal Programming and Hydroelectrolyte Metabolism Laboratory, Nucleus of Medicine and Experimental Surgery, Department of Internal Medicine, FCM, Campinas State University (UNICAMP), Campinas, SP Brazil

**Keywords:** DOHaD, Maternal protein restriction, Kidney, Metabolic programming, Early life, Long-term risk

## Abstract

Chronic kidney disease is an increasing global public health concern, and the Developmental Origins of Health and Disease (DOHaD) concept proposes that adverse conditions during critical developmental windows predispose offspring to chronic disorders later in life. Maternal protein restriction (MPR), a well-established experimental model reflecting food insecurity, has been shown to impair nephrogenesis and promote long-term renal dysfunction. In this study, we investigated renal metabolic–epigenetic programming induced by gestational and lactational MPR in post-weaning male rats using a global kidney proteomic approach. MPR altered renal structure and profoundly dysregulated protein networks, characterized by downregulation of energy metabolism, ion transport, cytoskeletal organization, membrane integrity, and mitochondrial function, alongside upregulation of innate immune pathways, glutathione metabolism, vesicular trafficking, and cytoskeletal dynamics. Integrated pathway and disease enrichment analyses revealed the potential risk to hypertension, acid–base imbalance, renal tubular transport disorders, nephrosis, and renal failure. Key differentially expressed proteins (e.g., GPX1, CYCS, ATP1A2/ATP1B1, TUBB/TUBA isoforms, ANPEP, and metabolic enzymes) emerged as potential biomarkers of renal metabolic–epigenetic programming. Collectively, these findings identify molecular signatures that link early-life protein restriction to long-term risk of kidney disease and provide mechanistic insight into the nephron- and cell-specific consequences of MPR.

**Supplementary Information:**

The online version contains supplementary material available at 10.1007/s13105-026-01189-9.

## Introduction

 In the 1980s, epidemiologist David Barker, through studies with pregnant women and newborns, showed that exposure to stressors in the maternal intrauterine environment can have an impact on adult health, increasing the risk of metabolic diseases [1]. This hypothesis gave rise to the Developmental Origins of Health and Disease (DOHaD) line of research, which associates the perinatal environment with embryonic/fetal development, a critical period vulnerable to adverse environmental insults [2].

The current situation is quite alarming. Epidemiological data from the World Health Organization (WHO) classify maternal hunger as a significant public health issue. One in 11 people worldwide faced hunger in 2023 (approximately 733 million people), according to the latest report on the State of Food Security and Nutrition in the World. In this context, the data warns that the world is significantly behind on Sustainable Development Goal 2, Zero Hunger, by 2030 [3]. The report shows that the world has regressed 15 years, with undernutrition levels comparable to those of 2008–2009. Although progress has been made in increasing exclusive breastfeeding rates among infants to 48%, achieving global nutrition targets will be a challenge. The prevalence of low birth weight has stagnated at around 15%, and stunting among children under five, although decreasing to 22.3%, still falls short of targets [3].

Accordingly, the International Society of Nephrology recognizes and addresses, with concern, the growing and alarming burden of kidney disease, especially in low- and middle-income countries. It is noteworthy that, for the first time, kidney health has been formally prioritized in the WHO’s noncommunicable diseases (NCD) agenda, paving the way for early detection, better prevention, improved access to treatment, and stronger health systems, in alignment with the United Nations Sustainable Development Goals 3.4 and 3.8 [4].

One of the most widely used models to mimic the hungry context is Maternal Protein Restriction (MPR), which can lead to various problems for the rat’s offspring, such as an increased incidence of prostate cancer and activation of oncological pathways, a lower number of β-cells and decreased insulin production, as well as hypertension and cardiac alterations [5–8]. In addition, low birth weight, associated with gestational MPR, decreases the number of nephrons by 28%, reduces salt excretion, and can lead to chronic renal failure and increased systolic blood pressure in adulthood [9–14]. In rats, nephrogenesis continues until around the twelfth day of life, and if the MPR is prolonged during the breastfeeding period, the reduction in the number of nephrons is 38% [14]. The impact of MPR on kidney development, such as nephrogenesis, protein synthesis, tissue remodeling, and cell fates of different kidney progenitor cells, can be intrinsically linked to the development of chronic kidney disease (CKD) and hypertension. There is currently an epidemic of CKD, posing a major public health challenge worldwide [15, 16], being a relevant cause of morbidity and mortality in Brazil and worldwide, which, according to the WHO, affects around 10% of the population, with an exponential increase.

Therefore, we aimed to evaluate the consequences of MPR on the renal proteomic landscape of male rats early in life. Given the effects of MPR established in the literature - such as hypertension - we hypothesize that there are essential proteins and signaling pathways impacted in the kidneys early in life, which have the potential to be biomarkers for not only immediate, but also medium- and long-term disorders, characterizing the risk of developmental origins of kidney health or disease.

## Materials and methods

### Experimental design

The experimental procedures followed the Ethical Principles in Animal Experimentation adopted by the Brazilian Society of Science in Laboratory Animals (SBCAL). They were approved by the Ethics Committee on the Use of Animals of the Institute of Biosciences of Botucatu (CEUA No 5119280121). Efforts were made to minimize suffering and reduce the number of animals used in the experiments. The acquisition and description of data followed the recommendations set out in the ARRIVE guidelines.

Adult males and females *Sprague Dawley* rats (90 days old; *n* = 40) were purchased from the State University of Campinas’ Central Stock Breeder (Campinas, SP, Brazil). The rats were maintained in a temperature-controlled environment (22–25 °C), relative humidity (55%), and photoperiod (12 h/12 h), with free access to water and food. Breeding occurred overnight in a harem (1 male: 3 females). Immediately following pregnancy confirmation (gestational Day 1 - GD1) by detecting spermatozoa in a vaginal smear, pregnant rats were divided into two experimental groups: (1) Control (CTR, *n* = 20), which received a standard diet (17% protein) during gestation and lactation, and (2) Gestation and Lactation Low Protein Diet (GLLP, *n* = 20), which received a low protein diet (6% protein) during gestation and lactation – Supplementary Table 1 [17]. PragSoluçoes (PragSoluçoes, SP, Brazil) supplied the isocaloric and normosodic diets. Water, food intake, and dam body mass were daily measured during pregnancy and lactation.

The male rat pups were euthanized by an overdose of ketamine and xylazine, followed by decapitation at postnatal day (PND)21 (Experimental design – Supplementary Fig. 1). The age was selected as it marks the end of lactation, allowing for the assessment of the immediate and initial effects of MPR on the offspring. Kidneys were extracted and weighed before being fixed and processed using the histological procedures (*n* = 5/group from different litters) described below. Other kidneys were promptly frozen in liquid nitrogen and stored at -80 °C (*n* = 5/group from different litters).

### Histological analysis

These analyses were based on Fioretto et al., 2025 [18]. Sections of kidney (Paraplast Plus^®^; 5 μm) were stained with hematoxylin-eosin (HE) to study the general structure and morphometric measurement (*n* = 5) or with picrosirius red for collagen detection (*n* = 5). Histological sections were analyzed in a Leica DMLB 80 microscope (Leica, Germany) coupled with the Leica Q-win software (Version 3 for Windows). The glomeruli area [A = π · a (semi-major axis). b (semi-minor axis)] and collagen content in both organs were determined in 10 histological sections per animal (*n* = 5/group), a total of 50 measurements/group. Morphometric data were obtained using ImageJ software (Version 1.45, NIH, Bethesda, Maryland, USA).

### Proteomic analysis by mass spectrometry

#### Extraction, digestion, and protein proteolytic cleavage

Global proteomic profiling was performed to investigate differentially expressed proteins (DEP) in the kidneys of rats exposed to MPR to contrast impacts at PND21 – early life. The kidney samples (100 mg; *n* = 5/group) from CTR and GLLP animals were processed in 1000µL of extraction buffer (8 M Urea, 1 M Tris, 100 mM PHSF, protease inhibitor, 65 mM DTT). The homogenate was ultrasonicated with ultrapure water (4 °C for 5 min) and centrifuged at 4 °C (30 min at 14.000 rpm) three times. The supernatant was collected, and the total protein quantification was determined by the Bradford Test, in duplicate [19]. The mass spectrometry and protein expression were performed according to Naia Fioretto et al. (2024) [20]. The kidney samples (200 µg of protein) were diluted (50 µL of ammonium bicarbonate) and digested. The proteins were reduced with dithiothreitol, alkylated with iodoacetamide, and digested with trypsin (16 h). The reaction was halted with trifluoroacetic acid, followed by centrifugation and desalting using Sep-Pak C18 cartridges (Waters, Milford, USA). The solutions were reduced by means of vacuum centrifugation, and subsequently the peptides were analyzed by liquid chromatography tandem mass spectrometry (LC–MS/MS).

#### Protein profile determination

The analyses were conducted according to Naia Fioretto et al., 2024 [20]. Peptide analysis was performed using the nanoAcquity UPLC Xevo QTOF G2 MS system (Waters, Manchester, UK). The results were analyzed with ProteinLynx Global Server (PLGS) software, identifying Rattus norvegicus proteins by the UniProt database and Monte Carlo algorithms. For the quantitative analysis of the proteome by PLGS software, only proteins with a confidence score > 95% were considered. In the comparative study between the groups (GLLP versus CTR at PND21), the proteins were selected as Differentially Expressed Protein (DEP), considering negatively regulated (downregulated) when *p* ≤ 0.05 (or when unique in the CTR group) and proteins positively regulated (upregulated) when *p* ≥ 0.95 (or when unique in the GLLP group). The cutoffs of *p* ≤ 0.05 and *p* ≥ 0.95 were used to increase the reliability of the study, increasing the significance and degree of expression of the different proteins. The raw data from the proteomic analyses are available in the Supplementary Material (Supplementary File 1) for further studies.

#### Proteomic analysis statistics

The identification of cellular and biological functions was conducted using Gene Ontology (GO) classification. Protein analysis was further refined through the Protein Annotation Through Evolutionary Relationship (PANTHER) database (http://pantherdb.org/). Protein codes were identified in the UniProt (https://www.uniprot.org/) and converted into gene names (Gene ID conversion tool).

#### Functional annotation analysis and data representation: *In silico* approach

##### a Data Selection and Integration

In the proteomic analyses at PND21, we found 108 differentially expressed proteins (DEP – excluded proteins with OK value 0 and out of *p* ≤ 0.05 or *p* ≥ 0.95) in the kidneys (43 upregulated and 65 downregulated - Supplementary File 1). We accessed the KOBAS-i 3.0 tool (http://kobas.cbi.pku.edu.cn/) to identify the top 12 biological pathways upregulated and downregulated enriched by DEP, using databases KEGG, Reactome, Panther, and Gene Ontology (Supplementary Files 2 for downregulated and 3 for upregulated) [21]. Bar plots (contingency charts) were created using GraphPad Prism^®^ software, converting the corrected p-value to -Log10 (version 8.0.1, Graph Pad, Inc., San Diego, CA).

##### b General DEP enrichment of pathways, biological, epigenetic, and potential diseases

Furthermore, we performed the most significant ontological enrichment which all DEP proteins (downregulate and upregulated separated) of 10 cellular processes (GO Cellular Component 2025), 10 molecular processes (GO Molecular Function 2025), and 10 enriched metabolites (Misc HMDB metabolites) on the Enrichr platform (https://maayanlab.cloud/Enrichr/) [22–24], considering the p-value significance (Supplementary Files 4, 5, and 6 for downregulated and Supplementary Files 7, 8, and 9 for upregulated). For cellular and molecular processes, we used *Appyter software* (https://appyters.maayanlab.cloud/#/) for graph construction, and considered the -Log10(p-value)/Odds Ratio and UMAP in the graphics, respectively.

From all the enrichment, we performed protein-protein integration in the STRING platform (https://string-db.org/) [25], using multiple proteins for *Rattus norvegicus*, with the following parameters: Full string network, 0.700 high confidence, to investigate the major interactions between proteins. After that, we selected only proteins with protein-protein interactions to investigate the epigenetic potential of regulating these DEPs. Subsequently, we investigated the validated and predicted microRNAs in the MirWalk platform (http://mirwalk.umm.uni-heidelberg.de/) [26], selecting the analysis for rat (Supplementary File 10). The interaction graphs were constructed in the SankeyMATIC platform (https://sankeymatic.com/).

To investigate the associations between our genes of interest and human diseases, we performed a functional enrichment analysis using gene sets from the DISEASES database (https://maayanlab.cloud/Harmonizome/resource/DISEASES), using the 35 DEP with epigenetic potential deregulation. This database integrates evidence of associations between genes and diseases from multiple sources, including: (1) Disease gene evidence scores by manual literature curation; (2) Disease gene evidence scores by integrating experimental data (GWAS); and (3) Gene-disease co-occurrence scores from text-mining biomedical abstracts. The analysis was conducted in the R environment version 4.3.0 [27], using the clusterProfiler [28, 29], GSEABase [30], and tidyverse [31] packages for data processing and analysis. Statistical significance was assessed using the Benjamini-Hochberg method to correct for multiple testing, with a significance threshold of *p* < 0.05. The chord diagram was created using the circlize package [32]. A BibTeX entry for LaTeX users is @Manual{, title = {R: A Language and Environment for Statistical Computing}, author = {{R Core Team}}, organization = {R Foundation for Statistical Computing}, address = {Vienna, Austria}, year = {2022}, url = {https://www.R-project.org/},}. The circos plot was constructed based on statistical analysis of binary distance for the dendrograms (Supplementary File 11).

##### c Association between DEP and potential kidney disorders associated with kidney injury data sets and epigenetic potential

Separately, we integrated our data using all upregulated and downregulated DEPs with two kidney disease databases: nephron-deficit CKD in the HSRA rat model ( availablein Milner et al., 2025 - Significantly Altered Protein Groups: No Diet Injured vs. Healthy Kidney Lysates − 120 min Gradient) [33] and ischemic AKI models in mice [34] (Differential Proteins) (Supplementary File 12). From this, we created the interaction graph in the Venny 2.1 platform (https://bioinfogp.cnb.csic.es/tools/venny/) [35]. We constructed the degrees of expression in a heat map graph, on the ChiPlot platform (https://www.chiplot.online/) – considering the different statistical cuts in each study – LogFC [33], p-value for our model, and Log2 Ratio [34] – highlighting all DEP commonly deregulated between the models and their degree of expression – upregulated or downregulated.

##### d Organ and tissue-specific single-cell analyses associating the main interactions of DEP and potential biomarkers of renal programming

Subsequently, we contrasted the epigenetic enrichment performed on the miRWalK platform (35 DEP) with that from the integration with disease datasets (49 DEP), observing a total of 17 DEP - represented by the Venny 2.1 platform (https://bioinfogp.cnb.csic.es/tools/venny/) [35]. To better elucidate the DEPs, we represented, from the STRING platform (https://string-db.org/) [25], the full STRING network interaction, high confidence (0.7) for *Rattus norvergicus*, observing the formation of 5 clusters (15 DEP). Finally, we accessed the Kidney Cell Explorer platform (https://cello.shinyapps.io/kidneycellexplorer/) based on Single-Cell Profiling Regional Diversity in the Mouse Kidney by RNAseq datasets [36], from the normalized Log expression normalization values, with average expression (rescaled) to visualize the expression of the 15 DEP in the nephron and ureteric epithelium, vasculature, immune and interstitial cells, in addition to nephron-ureteric-specifically region expression.

## Statistical analysis

Statistical analyses were performed using GraphPad Prism^®^ software (version 8.0.1, GraphPad, Inc., San Diego, CA) for the histological analysis. The Shapiro-Wilk test was used to test the normality of the data. The parametric data were submitted to the Student’s t-test, and the non-parametric data were evaluated by Mann-Whitney. Data were expressed as mean± standard deviation (SD). Differences were considered statistically significant when *p* < 0.05.

## Results

In the biometric parameters of offspring at PND21, we observed decreased body mass, length, and kidney mass (Table 1).

**Table 1:** Biometric parameters of offspring at PND21

Parameters	CTR	GLLP
Body Mass at PND21 (g)	46.4 ± 10.1	20.4 ± 4.1^* *p*=0.0140^
Length (cm)	19.3 ± 0.9	13.8 ± 1.4^* *p*<0.0001^
Kidney Mass (g)	0.3 ± 0.06	0.1 ± 0.01* p<0.0001

*Statistically significant difference between control (CTR) and gestational and lactational protein restriction (GLLP). Data are presented as mean ± SE (*n* = 5/group). Statistical analysis by the “t” test, with significance when p-value < 0.05.

**Fig. 1:**
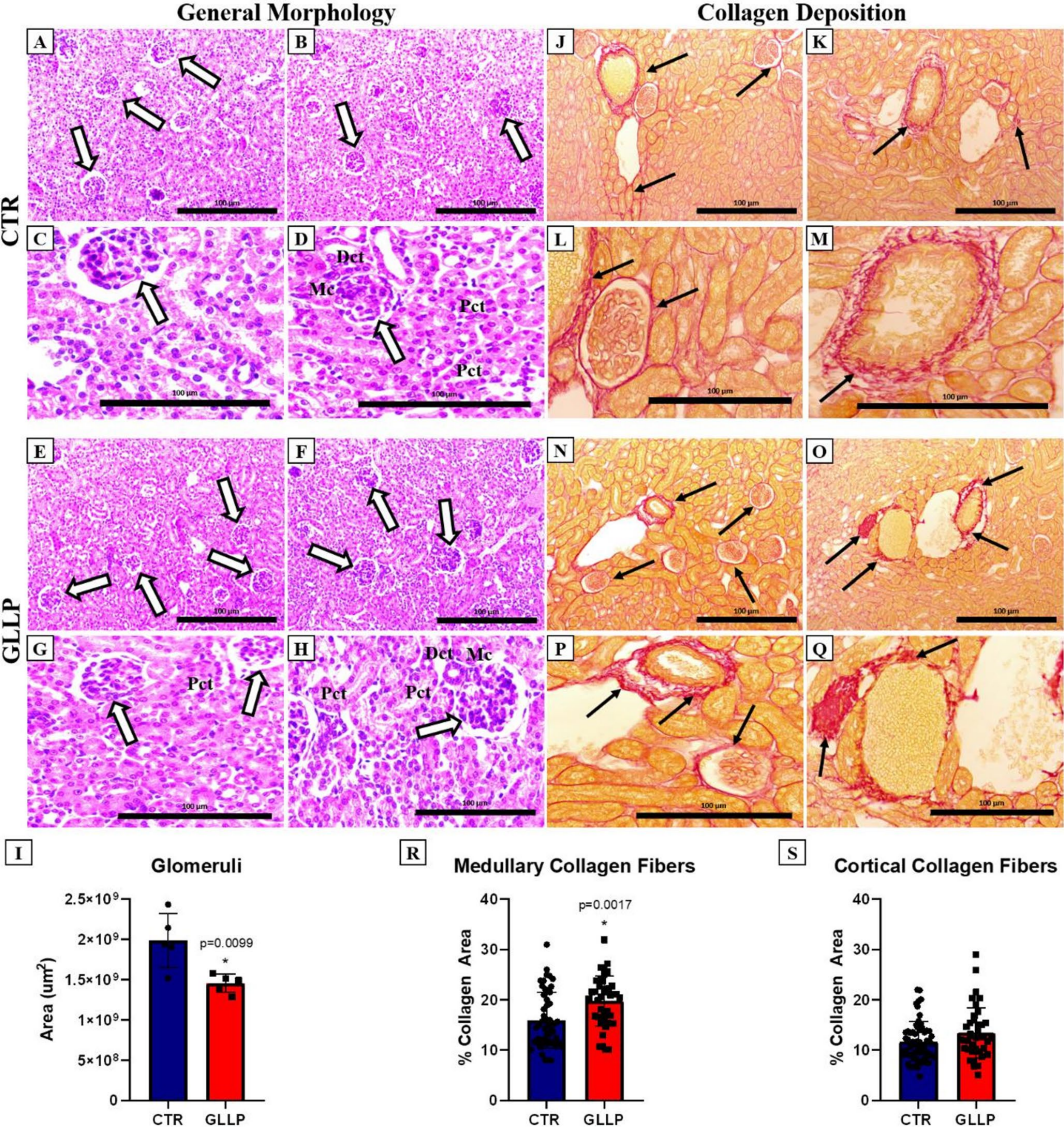
Morphological and morphometric analyses of the kidneys of CTR and GLLP animals. Representative histological sections stained with hematoxylin and eosin from the CTR (A-D) and GLLP (E-H) groups. White arrows indicate the glomeruli. I. Quantification of the glomeruli area. Representative histological sections stained with the Picrosirius histochemical technique from the CTR (J-M) and GLLP (N-Q) groups. Black arrows indicate collagen deposition. R. Quantification of the total collagen area in the medullary and cortical region (S).Mc. Macula densa. Dct. Distal convoluted duct. Pct. Proximal convoluted duct. Data are expressed as mean ± SD (*n* = 5/group). Statistical difference between experimental groups with *p* < 0.05 (*) or *p* < 0.0001 (**), tested using normality with the Shapiro-Wilk and Student’s t-test

The phenotypic results demonstrate that MPR leads to a decrease in the glomeruli area (Fig. 1A-I), in addition to an increase in the total collagen area in the medullary region; however, without differences in the cortical region (Fig. 1J-R).

**Fig. 2:**
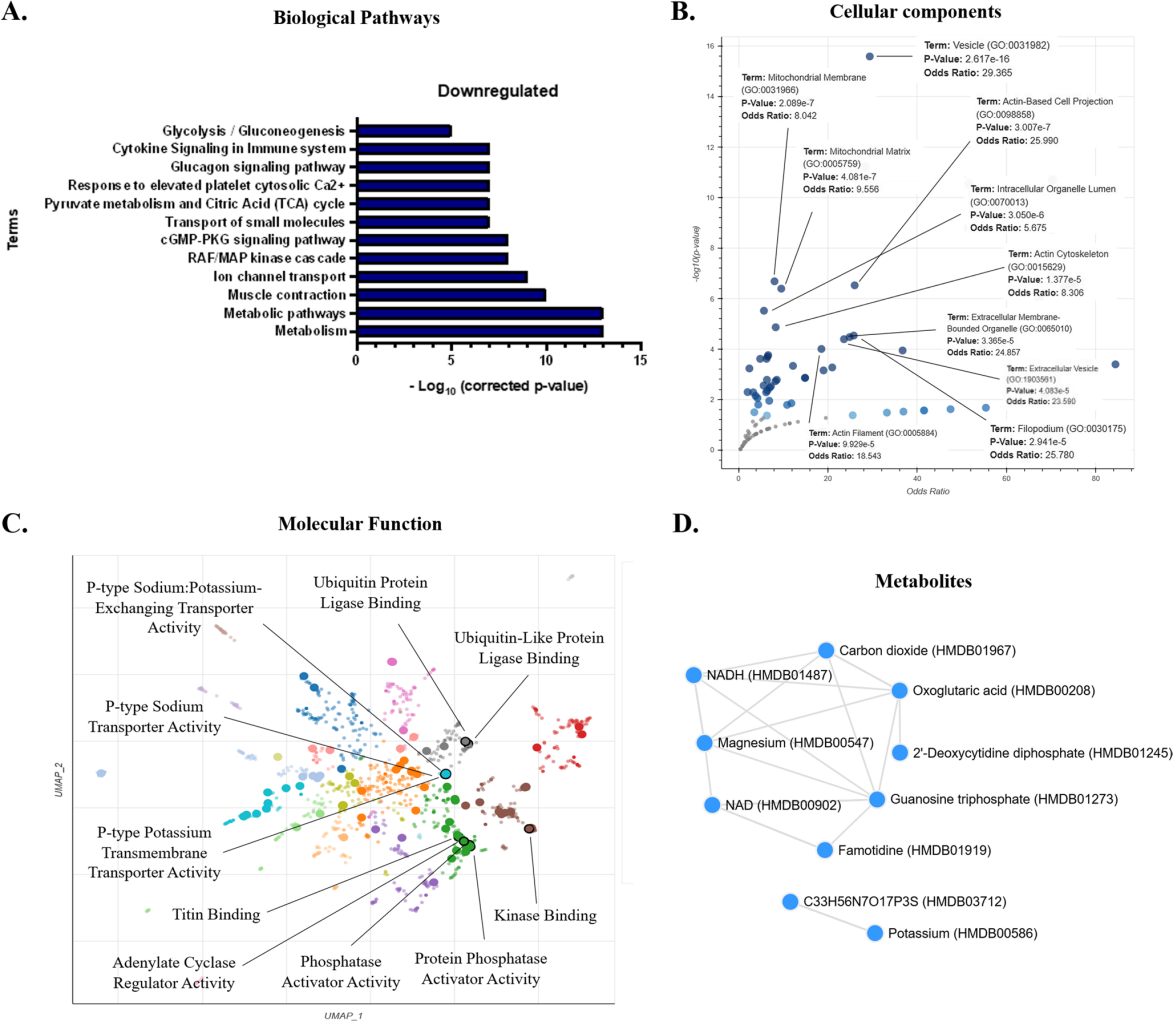
General biological enrichment of the 65 downregulated DEP. (**A**) Enrichment of 12 biological pathways elucidating aspects of cellular control, intracellular transport, muscle contraction, and metabolic disorders. Data were obtained from the KOBAS 3.0 platform, using the Panther, Reactome, GO, and KEGG databases. (**B**) Enrichment of cellular processes associated mainly with structural components. Analyses were performed on the Enrichr platform, using the GO Cellular Process 2025 database. (**C**) Enrichment of molecular processes, elucidating the 10 main processes. Scatterplot of the analyses performed on the Enrichr platform, with the GO Molecular Process 2025 database. Each point represents a term in the library. Term frequency-inverse document frequency (TF-IDF) values ​​were computed for the gene set corresponding to each term, and UMAP was applied to the resulting values. Terms are plotted based on the first two dimensions of UMAP. Terms were automatically identified with the Leiden algorithm. (**D**) Plot of terms from the HMDB_Metabolites gene set analyzed in the Enrichr platform. Statistical analyses associated with the corrected p-value and -Log10 of the corrected p-value (**A**), p-value (**B, D**), and p-value and TF-IDF (**C**)

**Fig. 3:**
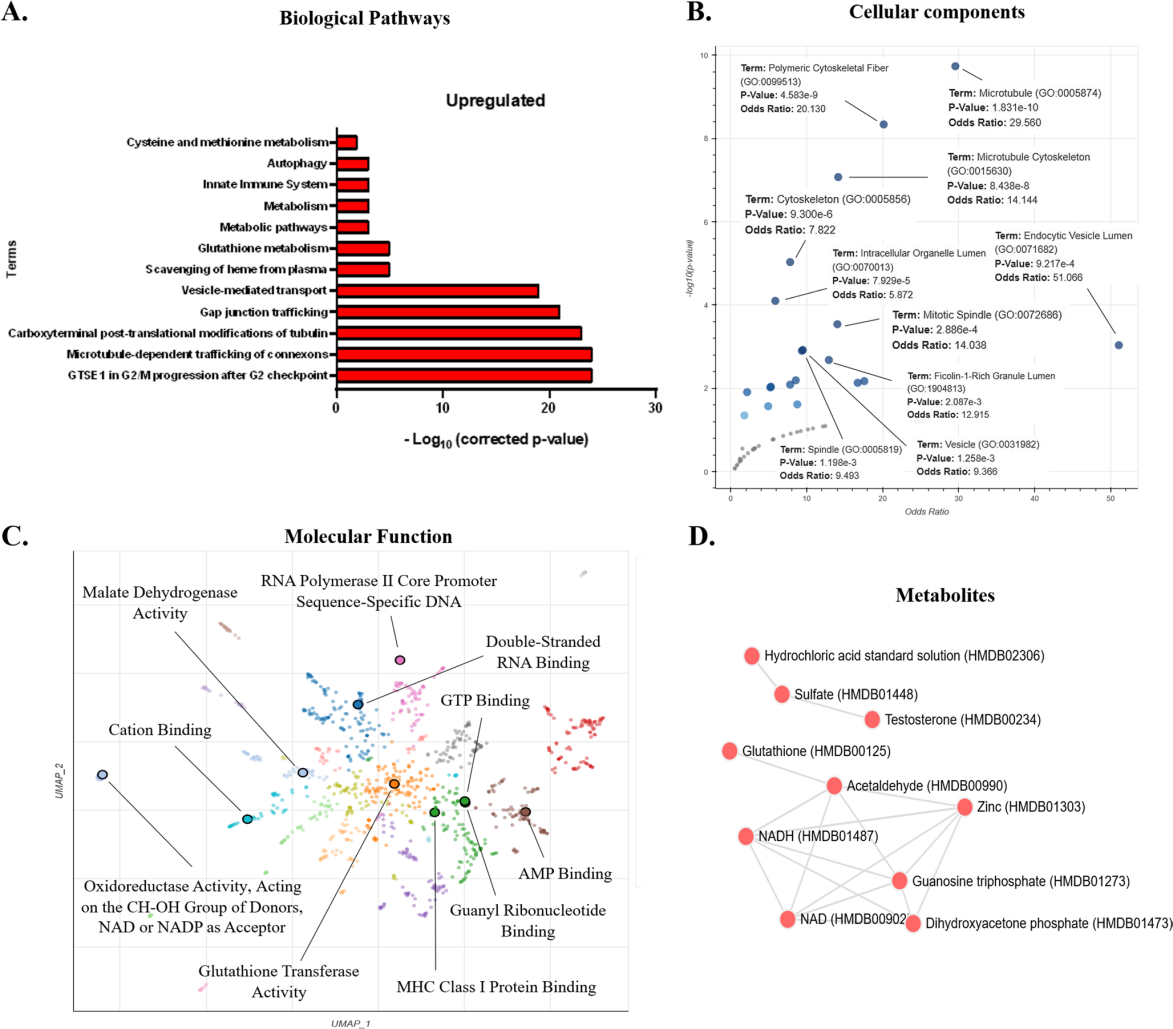
General biological enrichment of the 43 upregulated DEP. (**A**) Enrichment of 12 biological pathways elucidating aspects of cellular control, intracellular transport, muscle contraction, and metabolic disorders. Data were obtained from the KOBAS 3.0 platform, using the Panther, Reactome, GO, and KEGG databases. (**B**) Enrichment of cellular processes primarily associated with structural and cytoskeletal aspects. Analyses were performed on the Enrichr platform, using the GO Cellular Process 2025 database. (**C**) Enrichment of molecular processes, elucidating the 10 main processes. Scatterplot of the analyses performed on the Enrichr platform, with the GO Molecular Process 2025 database. Each point represents a term in the library. Term frequency-inverse document frequency (TF-IDF) values ​​were computed for the gene set corresponding to each term, and UMAP was applied to the resulting values. Terms are plotted based on the first two dimensions of UMAP. Terms were automatically identified with the Leiden algorithm. (**D**) Plot of terms from the HMDB_Metabolites gene set analyzed in the Enrichr platform. Statistical analyses associated with the corrected p-value and -Log10 of the corrected p-value (**A**), p-value (**B, D**), p-value and TF-IDF (**C**)

Proteomic analyses of kidneys at PND21 elucidated 108 DEPs between the CTR and GLLP groups. Of these, 65 were downregulated, while 43 were upregulated in the GLLP group. From the enrichment of biological pathways, we observed some downregulated pathways associated with muscle contraction, transport of small molecules, pyruvate metabolism, and citric Acid (TCA) cycle (Fig. 2A); and upregulated pathways associated with cell cycle, microtubules, vesicle-mediated transport, and immune system (Fig. 3A).

Regarding cellular components, we observed several downregulated components associated with vesicles, mitochondria, and cytoskeleton (Fig. 2B); and upregulated components associated with microtubule, cytoskeleton, and endocytic vesicle lumen (Fig. 3B). Regarding the downregulated molecular aspects, our results demonstrated ubiquitin protein ligase binding, sodium and potassium transporter activity (Fig. 2C); and upregulated associated to GTP binding, guanyl ribonucleotide binding, and oxidoreductase activity (Fig. 3C). Finally, we verified some metabolites enriched in a downregulated manner, such as NADH, NAD, and carbon dioxide (Fig. 2D), and upregulated, such as guanosine triphosphate, glutathione, NADH, and NAD (Fig. 3D).

**Fig. 4:**
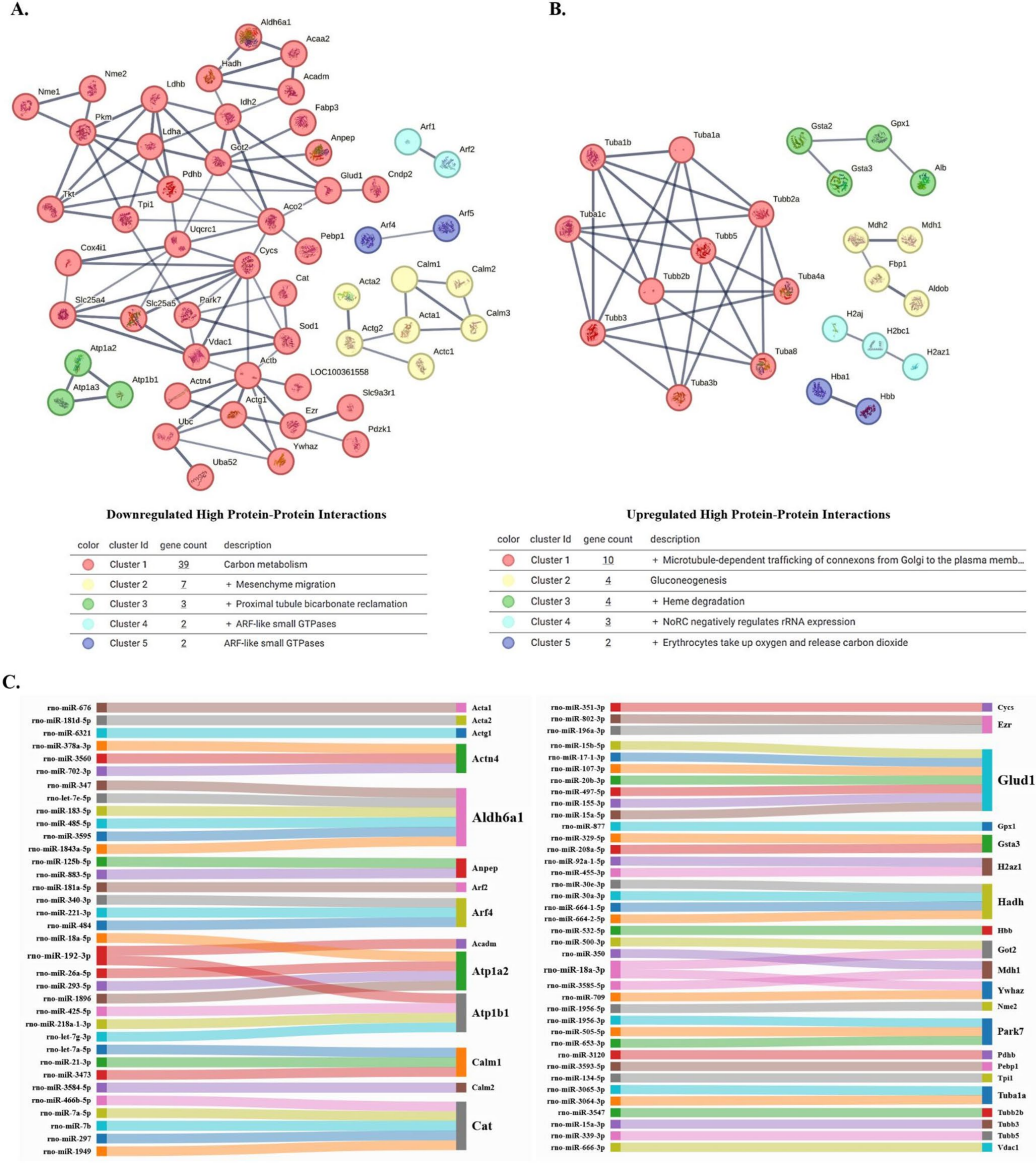
Protein-protein interaction network between downregulated (**A**) and upregulated (**B**) DEP at PND21 and microRNAs (miRNAs) target as epigenetic modulation (**C**). Interactions of the identified proteins were mapped by searching the STRING database with a confidence cut-off of 0.7. In the resulting protein association network, proteins are presented as nodes that are connected by lines whose thickness represents the confidence level (0.7) (**A** and **B**). The Sankey diagram illustrates the regulatory relationships, where each colored ribbon represents the connection of one or more miRNAs to their corresponding gene targets. Several genes are regulated by multiple miRNAs, highlighting their potential as central nodes in post-transcriptional regulation. This integrative approach reveals candidate biomarkers and molecular pathways potentially involved in metabolic and stress-response processes. The analysis was performed in the mirWalk tool, and the graphics were constructed in the SankeyMatic (**C**)

Subsequently, we performed the specific protein-protein interaction between the downregulated (Fig. 4A) and upregulated (Fig. 4B) DEP. In the downregulated DEP, we observed 5 clusters, associated with Carbon metabolism, Mesenchyme migration, Proximal tubule bicarbonate reclamation, ARF-like small GTPases, and ARF-like small GTPases (Fig. 4A). In the upregulated DEP, we observed 5 clusters, associated with Microtubule-dependent trafficking of connexons from Golgi to the plasma membrane, Gluconeogenesis, Heme degradation, NoRC negatively regulated rRNA expression, and Erythrocytes take up oxygen and release carbon dioxide (Fig. 4B).

Potential epigenetic interactions revealed multiple miRNA–target associations involving key genes related to cytoskeleton (e.g., *Acta1/2*,* Actg1*,* Tuba1a*,* Tubb isoforms*), metabolism (e.g., Aldh6a1, Glud1, Mdh1, Pdhb, Tpi1), ion transport (Atp1a2, Atp1b1), redox balance (Cat, Gpx1, Gsta3), and mitochondrial function (Cycs, Vdac1, Hadh). These targets were regulated by a diverse set of miRNAs (e.g., rno-miR-21-3p, let-7 family, miR-134-5p, miR-181 family), suggesting broad epigenetic modulation of metabolic, structural, and cellular homeostasis pathways (Fig. 4C).

In addition, we observed the correlation between these DEP associated with epigenetic potential and risk for metabolic-renal diseases. We observed the enrichment of organic acidemia, hypothyroidism, protein-energy malnutrition, dilated cardiomyopathy, nephrosis, lactic acidosis, 2-hydroxyglutaric aciduria, bilirubin metabolic disorder, essential hypertension, metabolic acidosis, gamma-amino butyric acid metabolism disorder, succinic semialdehyde dehydrogenase deficiency, urea cycle disorder, renal tubular transport disease, and acute kidney failure (Supplementary Fig. 2).

**Fig. 5:**
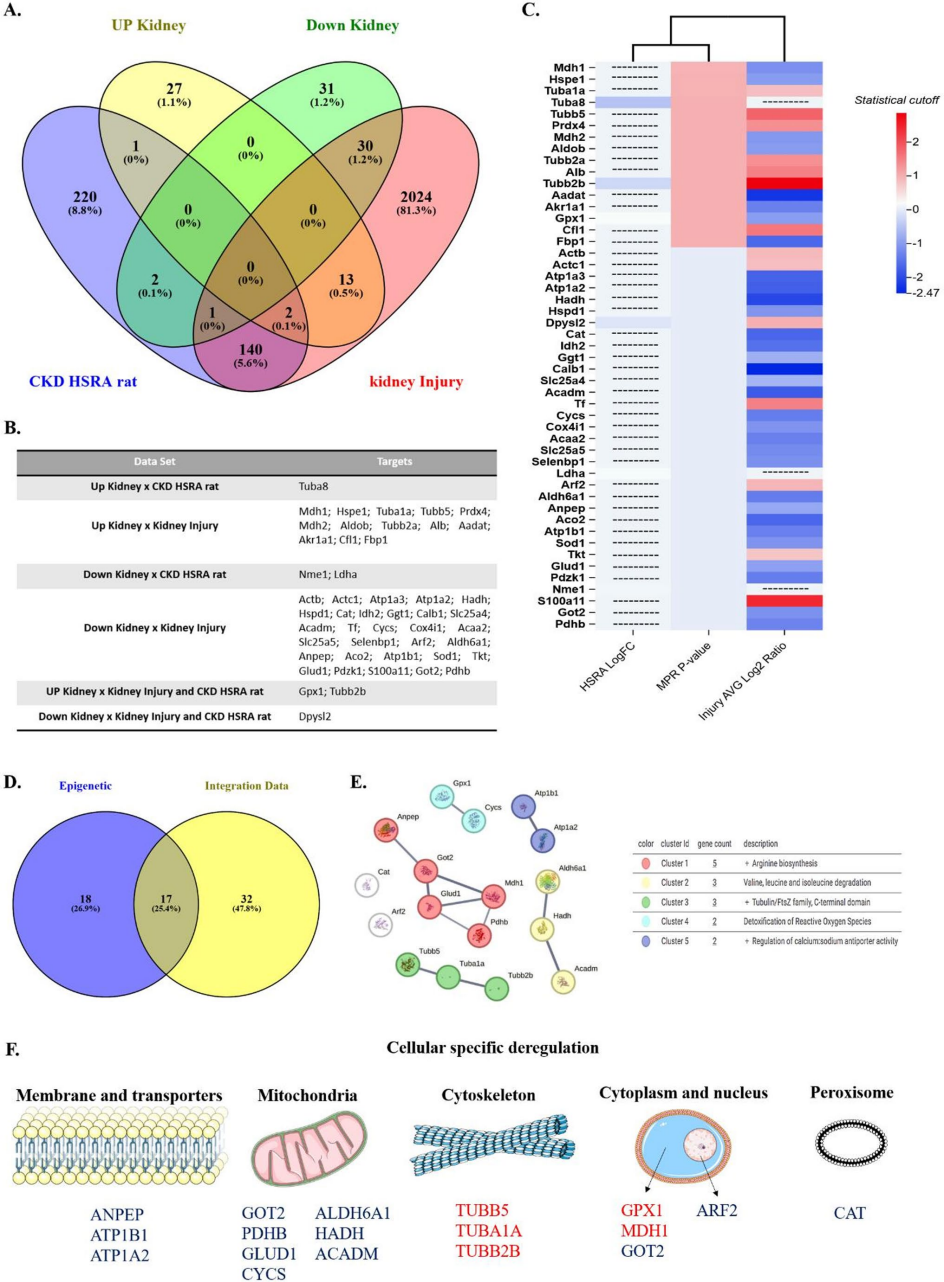
Integration of upregulated and downregulated proteomic data from the kidneys of rats exposed to MPR with proteomic datasets from chronic kidney disease single kidney (CKD HSRA) rats and kidney injury. **A**. Venny plot elucidating the common targets among the three models, highlighted in (**B**). **C**. Heat map constructed on the ChiPlot platform, elucidating the targets and the degrees of differential expression – adjusted p-value (MPR), LogFC (HSRA), and AVG Log2 Ratio (Injury). Integration obtained through public data available in Burton et al., 2024^34^ and Milner et al., 2025^33^. **D**. Venny plot demonstrating the 17 DEPs commonly dysregulated between the two analyses. **E**. Protein-protein interactions elucidating the clusters formed by parts of the strongest interactions (Arginine biosynthesis, valine, leucine, and isoleucine degradation, Tubulin/FtsZ family, C-terminal domain, Detoxification of reactive oxygen species, and Regulation of calcium: sodium antiporter activity). Analysis performed on the String platform. **F**. Organelle-specific expression of each DEP analyzed from the integrations, demonstrating the downregulated (blue) or upregulated (red) DEPs and the specific cellular regions where they are expressed (plasma membrane, mitochondria, cytoskeleton, cytoplasm, nucleus, and peroxisome). The Figure was composed using some illustrations from Servier Medical Art (https://smart.servier.com), licensed under CC BY 4.0 (https://creativecommons.org/licenses/by/4.0/)

Based on these investigations, we sought to understand possible correlations between the potential for renal DEP in early life and the risk of kidney disorders during adult and aging life, based on the kidney programming. Thus, we verified correlations with data sets of kidney injury and chronic kidney disease, in addition to correlating them with developmental aspects and general kidney disease (Fig. 5). We observed that, in common, there were the following correlations between Up Kidney x CKD HSRA rat, Up Kidney x Kidney Injury, Down Kidney x CKD HSRA rat, Down Kidney x Kidney Injury, UP Kidney x Kidney Injury and CKD HSRA rat, and Down Kidney x Kidney Injury and CKD HSRA rat (Fig. 5A, B), elucidating the degree of expression (down or up) through statistical cuts by the heat map (Fig. 5C).

We also integrated the common targets between epigenetic potential risk and the disease integration data (Fig. 5D-F). We found 17 DEPs commonly (Fig. 5D), and in the protein-protein interaction, we showed 5 clusters, including: Arginine biosynthesis (ANPEP, GOT2, GLUD1, MDH1, PDHB), Valine, leucine and isoleucine degradation (ALDH6A1, HADH, ACADM), Tubulin/FtsZ family, C-terminal domain (TUBB5, TUBA1A, TUBB2B0, Detoxification of reactive oxygen species (GPX1, CYCS), Regulation of calcium: sodium antiporter activity (ATP1B1, ATP1a2), and 2 targets without clusterization (CAT, ARF2) (Fig. 5E). The cellular specific deregulation in demonstrated in the Fig. 5F.

**Fig. 6:**
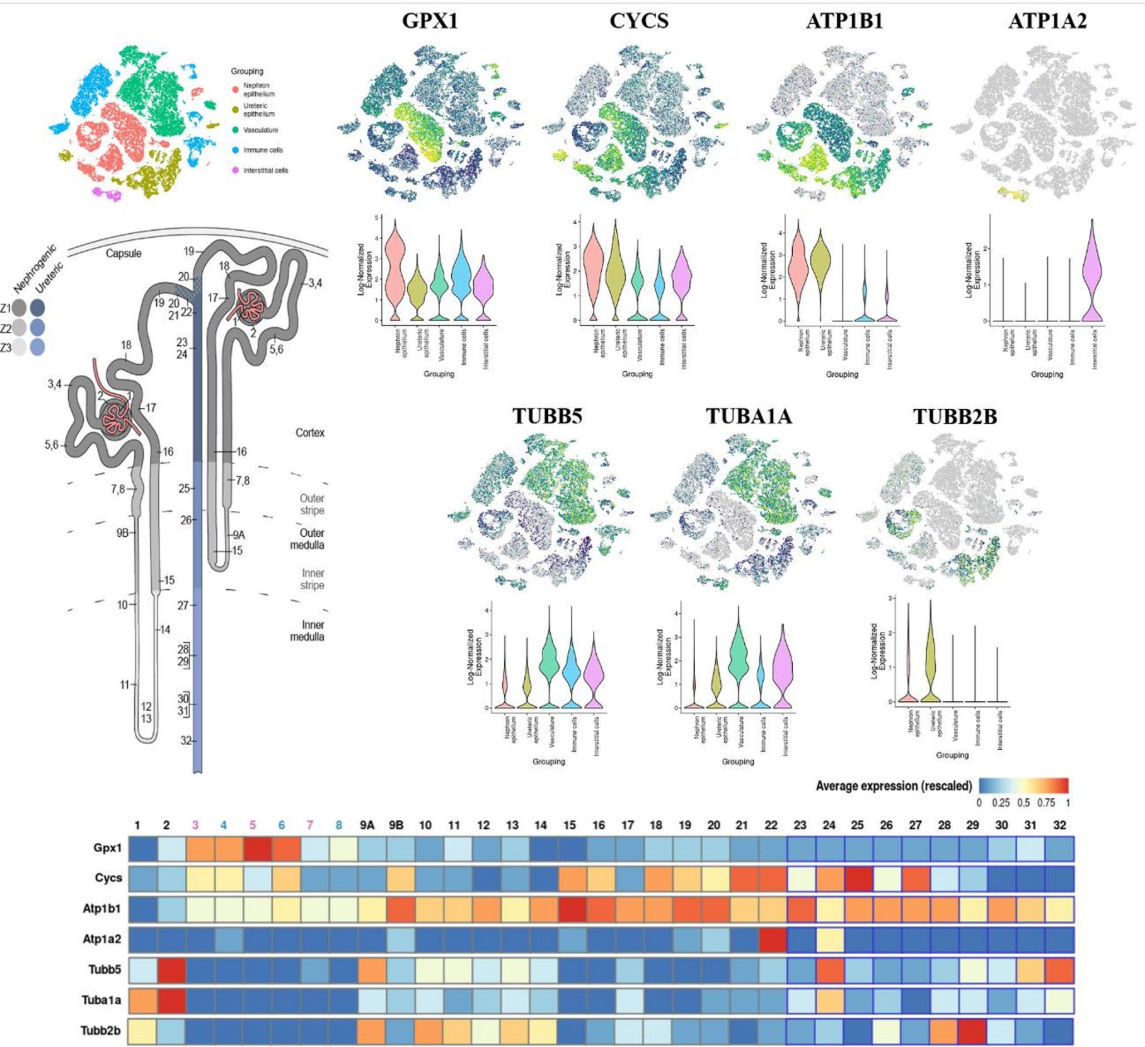
Single-cell analysis of GPX1, CYCS, ATP1B1, ATP1A2, TUBB5, TUBA1A, and TUBB2B targets in the kidneys and nephrons based on the selected DEPs dysregulated in GLLP animals. The map elucidating the general regions of the urinary system, a specific view of the functional units (nephrons), the most enriched and expressed regions in the urinary system, the graph plotting the most enriched and expressed regions in the urinary system, and the degree of expression of the seven DEPs specifically in the nephron and collecting duct cells are shown. Analysis performed on the Kidney Cell Explorer platform. Statistical analyses based on normalization values ​​of the normalized log expression, with mean expression (scaled). (1) Podocytes (visceral epithelium), (2) Parietal epithelium, (3) Proximal tubule segment 1 – female, (4) Proximal tubule segment 1 – male, (5) Proximal tubule segment 2 – female, (6) Proximal tubule segment 2 – male, (7) Proximal tubule segment 3 – female, (8) Proximal tubule segment 3 – male, 9 A. LOH thin descending limb of the inner stripe of the outer medulla of the cortical nephron, 9B. LOH thin descending limb of the inner strip of outer medulla of the juxtamedullary nephron, 10. Superior LOH thin descending limb of the inner medulla of the juxtamedullary nephron, 11. Inferior LOH thin descending limb of the inner medulla of the juxtamedullary nephron, 12. Inferior LOH thin limb of the inner medulla of the juxtamedullary nephron, 13. Inferior LOH thin limb of the inner medulla of the juxtamedullary nephron, 14. Superior LOH thin ascending limb of the inner medulla of the juxtamedullary nephron, 15. Distal straight tubule of inner strip of outer medulla (syn: thick ascending limb of LOH), 16. Distal straight tubule of outer strip of outer medulla and cortex (syn: thick ascending limb of LOH), 17. Macula densa, 18. Distal convoluted tubule, 19. Connecting tubule of nephron, 20. Principal-like cell of the connecting tubule of the nephron, 21. Intercalated type non-A non-B cell of the connecting tubule of the nephron, 22. Intercalated type A cell of the connecting tubule of the nephron and cortical collecting duct, 23. Principal-like cell of the cortical collecting duct, 24. Intercalated type B cell of the cortical collecting duct, 25. Intercalated type A cell of the outer medullary collecting duct, 26. Principal cell of the outer medullary collecting duct, 27. Intercalated type A cell of the inner medullary collecting duct, 28. Principal cell of the inner medullary collecting duct type 1, 29. Principal cell of the inner medullary collecting duct type 2, 30. Principal-like cell of the deep inner medullary collecting duct type 1, 31. Deep inner medullary collecting duct type 2 cell, 32. Deep medullary epithelium of the pelvis

Cluster analysis revealed nephron segment–specific and cell-type–specific expression patterns of DEPs. GPX1 was broadly expressed across nephron epithelium, vasculature, immune, and interstitial cells, with enrichment in the proximal convoluted tubule. CYCS showed a similar distribution, with higher expression in the distal convoluted tubule and collecting duct. ATP1B1 was predominantly expressed in the nephron epithelium along the loop of Henle to the collecting duct, whereas ATP1A2 was mainly localized to interstitial cells, with enrichment at the beginning of the collecting duct. Cytoskeletal proteins (TUBB5, TUBA1A) were mainly expressed in vasculature, immune, and interstitial compartments, with nephron enrichment in Bowman’s capsule, while TUBB2B was specifically expressed in the nephron epithelium of the distal collecting duct (Fig. 6).

**Fig. 7:**
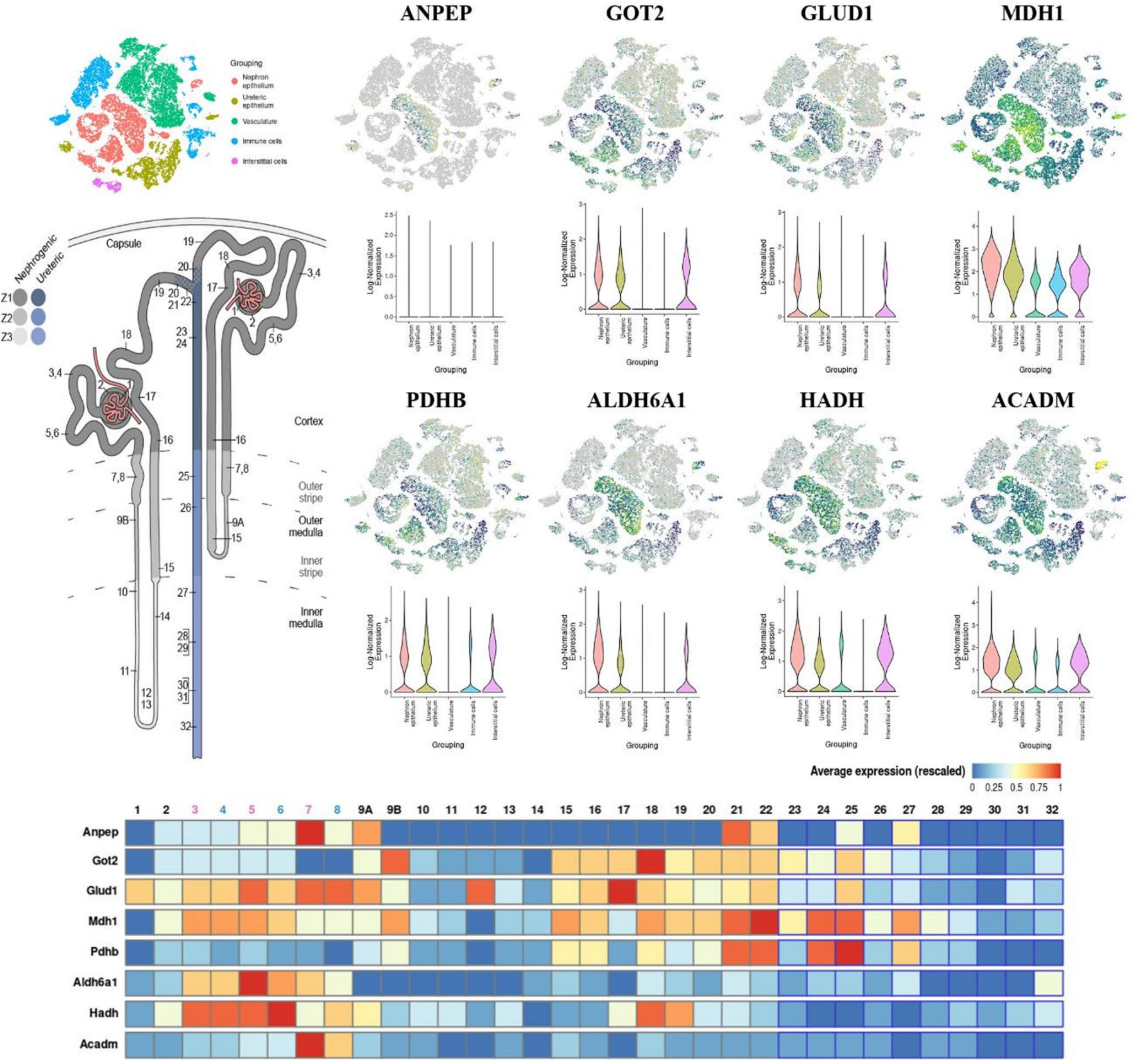
Single-cell analysis of ANPEP, GOT2, GLUD1, MDH1, PDHB, ALDH6A1, HADH, and ACADM targets in the kidneys and nephrons based on the selected DEPs dysregulated in GLLP animals. The map elucidating the general regions of the urinary system, a specific view of the functional units (nephrons), the most enriched and expressed regions in the urinary system, the graph plotting the most enriched and expressed regions in the urinary system, and the degree of expression of the seven DEPs specifically in the nephron and collecting duct cells are shown. Analysis performed on the Kidney Cell Explorer platform. Statistical analyses based on normalization values ​​of the normalized log expression, with mean expression (scaled). (1) Podocytes (visceral epithelium), (2) Parietal epithelium, (3) Proximal tubule segment 1 – female, (4) Proximal tubule segment 1 – male, (5) Proximal tubule segment 2 – female, (6) Proximal tubule segment 2 – male, (7) Proximal tubule segment 3 – female, (8) Proximal tubule segment 3 – male, 9 A. LOH thin descending limb of the inner stripe of the outer medulla of the cortical nephron, 9B. LOH thin descending limb of the inner strip of outer medulla of the juxtamedullary nephron, 10. Superior LOH thin descending limb of the inner medulla of the juxtamedullary nephron, 11. Inferior LOH thin descending limb of the inner medulla of the juxtamedullary nephron, 12. Inferior LOH thin limb of the inner medulla of the juxtamedullary nephron, 13. Inferior LOH thin limb of the inner medulla of the juxtamedullary nephron, 14. Superior LOH thin ascending limb of the inner medulla of the juxtamedullary nephron, 15. Distal straight tubule of inner strip of outer medulla (syn: thick ascending limb of LOH), 16. Distal straight tubule of outer strip of outer medulla and cortex (syn: thick ascending limb of LOH), 17. Macula densa, 18. Distal convoluted tubule, 19. Connecting tubule of nephron, 20. Principal-like cell of the connecting tubule of the nephron, 21. Intercalated type non-A non-B cell of the connecting tubule of the nephron, 22. Intercalated type A cell of the connecting tubule of the nephron and cortical collecting duct, 23. Principal-like cell of the cortical collecting duct, 24. Intercalated type B cell of the cortical collecting duct, 25. Intercalated type A cell of the outer medullary collecting duct, 26. Principal cell of the outer medullary collecting duct, 27. Intercalated type A cell of the inner medullary collecting duct, 28. Principal cell of the inner medullary collecting duct type 1, 29. Principal cell of the inner medullary collecting duct type 2, 30. Principal-like cell of the deep inner medullary collecting duct type 1, 31. Deep inner medullary collecting duct type 2 cell, 32. Deep medullary epithelium of the pelvis

In addition, ANPEP showed low overall renal expression but was enriched in the initial segment of the loop of Henle. GOT2 and GLUD1 were expressed in nephron epithelium and interstitial cells, with GOT2 enriched in the loop of Henle and distal convoluted tubule, and GLUD1 in the proximal tubule, loop of Henle, and distal tubule. MDH1 exhibited broad expression across renal compartments, with enrichment in the collecting duct. PDHB and ALDH6A1 were mainly localized to the nephron and ureteric epithelium and interstitial cells, with PDHB enriched in the collecting duct and ALDH6A1 in the proximal tubule. Finally, HADH and ACADM were expressed in nephron epithelium and interstitial cells, with HADH enriched in the proximal and early distal tubule, and ACADM in the initial descending loop of Henle (Fig. 7).

## Discussion

The major novelty of this work was to trace the immediate consequences of maternal exposure to a low-protein diet during pregnancy and lactation on the global proteomic profile of the kidneys of post-weaning male rats. MPR caused early renal structural alterations, including a smaller glomerular area and increased medullary collagen deposition. It is noteworthy that we elucidated numerous structural, cellular, biochemical, molecular, and epigenetic aspects, highlighting the potential risk of kidney disease and injury through the integration of in silico data. We compiled the main biomarkers and nephron- and cell-specific regions dysregulated in the kidneys during early life, establishing molecular and epigenetic signatures that may be disrupted or exacerbated in the medium and long term, increasing the risk of kidney disorders. MPR does not induce overt structural renal damage but disrupts key molecular processes, including energy metabolism, ion transport, mitochondrial function, and cytoskeletal organization. Concurrently, it activates adaptive responses such as glutathione metabolism, vesicular trafficking, and cell cycle regulation. These alterations are linked to epigenetic susceptibility and miRNA interactions, identifying potential biomarkers of nephron-specific dysfunction and chronic kidney disease risk.

Overall, the downregulated pathways in the kidneys of rats exposed to MPR revealed impairments in muscle contraction, energy metabolism, the tricarboxylic acid (TCA) cycle, and ion and small-molecule transport. In silico analyses further indicated mitochondrial and membrane dysfunction, along with disrupted electron and solute transport processes—which, in the proximal convoluted tubule, may signify substantial damage to this key site of reabsorption. Interestingly, Lamana et al. (2021) observed that, in adult life, these animals presented intensive sodium reabsorption in the nephron’s proximal segments [14]. Consistently, Wang et al. (2023) demonstrated that intrauterine protein malnutrition impairs kidney development by deregulating ciliogenesis, inhibiting β-catenin signaling, and promoting apoptosis in renal tubular epithelial cells [37]. Similarly, Guo et al. (2020) reported that MPR alters the expression of renal ion channels and transporters, decreasing aquaporins 2/4 and several solute carriers while upregulating others (Slc2a1, Slc4a1, Slc9a1, Slc29a3) [38]. MPR has been associated with increased urinary sodium loss and expanded extracellular fluid volume, supporting our findings of impaired ion transport [39–41]. Consistent omics evidence from other organs shows disrupted muscle contraction and altered energy metabolism [18, 20]. Together, these findings indicate that MPR impairs renal energy metabolism, contractility, and ion transport, potentially disrupting sympathorenal regulation and fluid balance, thereby increasing susceptibility to hypertension.

MPR upregulates pathways related to trafficking, vesicular transport, redox metabolism, and immune responses, with in silico analyses indicating disrupted cytoskeletal organization, endocytosis, and endoplasmic reticulum (ER)–Golgi dynamics. Supporting this, MPR during gestation has been shown to alter placental transcriptomic profiles, affecting cholesterol and lipoprotein metabolism and clathrin-mediated endocytosis [42], indicating that these may represent common organ-specific mechanisms of MPR exposure. MPR decreases AT receptor protein levels during nephrogenesis, followed by compensatory upregulation [40], consistent with adaptive transport responses also reflected in our findings. Furthermore, MPR has been shown to reduce nephron number and glomerular volume, with an increased glomerular filtration rate in adulthood following salt challenge [43]. Similarly, intrauterine growth restriction models revealed vascular and glomerular congestion, reduced endothelial nitric oxide synthase phosphorylation, induction of arginase-2, and elevated reactive oxygen species [44]. Overall, MPR triggers early adaptive activation of renal transport, metabolic, and redox pathways that may become maladaptive over time, promoting metabolic programming, oxidative imbalance, and early markers of renal dysfunction.

Integrating macroscopic to molecular findings, miRNA–DEP interactions highlight epigenetic regulation of key pathways, identifying potential biomarkers of early renal programming. The emergence of evolutionary developmental biology and DOHaD, grounded in *Hox* gene discovery, environmental stressors, and epigenetic regulation, provides essential insights into nephron development and lifelong kidney disease risk [45, 46]. Consistent with our findings, studies have shown that rats exposed to gestational MPR develop arterial hypertension and long-term hyperfiltration in adulthood, evidenced by proteinuria. These effects are linked to increased renal expression of miR-192 and the miR-200 family, promoting epithelial-to-mesenchymal transition through upregulation of TGF-β1, type I collagen, fibronectin, and ZEB [12]. Another example was miR-18a-3p, regulating Got2 and Ywhaz. The literature elucidates that male C57BL/6 mice and renal primary human cells treated with LPS (Lipopolysaccharide-Induced Acute Kidney Injury model) had pyroptosis with increased expression of lncRNA MEG3 in the renal tubular epithelium by regulating the miR-18a-3p/GSDMD pathway [47]. These data link epigenetic programming to renal alterations and CKD risk, highlighting potential biomarkers for future investigations.

In line with the enriched metabolic and renal disease pathways, and based on the associations between our identified markers and datasets related to chronic and injury-induced renal disorders, our findings suggest a potential renal programming toward long-term kidney dysfunction. Notably, we identified possible risk correlations with acidemia, protein–energy malnutrition, nephrosis, hypertension, urea cycle disorders, renal tubular transport diseases, and acute kidney injury, as well as convergence with biomarker profiles characteristic of chronic kidney disease and renal injury.

Burton et al. (2024) showed the renal proteomic landscape associated with acute kidney injury. Proteins downregulated in the injured kidney were involved in energy production, including numerous peroxisomal matrix proteins that function in fatty acid oxidation, such as ACOX1, CAT, EHHADH, ACOT4, and ACOT8 [34]. Milner et al. (2025) demonstrated that rats born with a single kidney and ~ 20% fewer nephrons—predisposed to CKD—already exhibited early epigenetic, transcriptomic, and proteomic alterations at 4 weeks of age, particularly involving NME1, LDHA, DPYSL2, GPX1, TUBB2B, and TUBA8, suggesting compensatory or developmental mechanisms underlying susceptibility to CKD [33]. Low birth weight is one of the consequences of MPR, and the literature explains that this condition can generate a tendency and risk for CKD and hypertension [48]. Experimental data support that MPR increases the risk of developing hypertension, a condition associated with disturbances in nephrogenesis [49] and renal response (e.g., renin-angiotensin-aldosterone system) [50]. A comparative study demonstrated that intrauterine growth restriction is a risk factor for hypertension and CKD, potentially resulting from exposure to MPR or maternal betamethasone treatment, both associated with reduced nephron numbers [51]. Although our findings suggest early-life disturbances that may predispose to renal metabolic disorders, long-term effects remain to be fully elucidated.

Integrative analysis identified 17 epigenetically associated DEPs (15 clustered), enriched in pathways related to amino acid metabolism, cytoskeletal organization, reactive oxygen species (ROS) detoxification, and calcium and sodium transport. Specifically, our results demonstrated renal impacts on energy metabolism and mitochondrial processes (GOT2, PDHB, GLUD1, HADH, ACADM, ALDH6A1, CYCS), structural impacts on the plasma membrane and transport (ANPEP, ATP1B1, ATP1A2), intracellular transport and cytoskeleton (TUBA1A, TUBB5, TUBB2B), cytoplasmic, antioxidant and enzymatic aspects (GPX1, MDH1, GOT2), peroxisome (CAT), and nucleus (ARF2) – specifying, including, the nephron-specific localization of the biomarkers (DEPs) present in each cluster.

Specifically, pyruvate dehydrogenase E1 (PDHB), a key mitochondrial enzyme linking glycolysis to the Krebs cycle via pyruvate-to-acetyl-CoA conversion, was downregulated in the MPR model, despite showing higher expression in the collecting duct. The literature correlates alterations in PDHB with clear cell renal carcinoma and papillary cell renal carcinoma [52, 53]. Glutamic-Oxaloacetic Transaminase 2 (GOT2), a key enzyme in amino acid metabolism, the TCA and urea cycles, and NAD(H) redox balance via the malate–aspartate shuttle, was downregulated, with predominant expression in the distal convoluted tubule. This molecule has also been associated with inflammatory aspects and clear cell renal carcinoma [54]. Glutamate dehydrogenase 1 (GLUD1) is a mitochondrial enzyme, essential for glutamate, nitrogen metabolism, and energy homeostasis, that catalyzes glutamate deamination to α-ketoglutarate and ammonia; it is highly expressed in early nephrons and distal tubules and was downregulated in our model. GLUD1 has been associated, in in vitro studies, with impaired renal response (oxidative injury and apoptosis) and possible liver damage [55]. Malate dehydrogenase 1 (MDH1) catalyzes the reversible conversion of malate to oxaloacetate as part of the malate–aspartate shuttle, and was overexpressed in our model (including MDH2), with predominant expression in the collecting duct. The literature does not correlate the direct effects of changes in MDH1, but has linked the inhibition of malate dehydrogenase-2 by MDH2 to the protection of renal tubular epithelial cells against anoxia-reoxygenation-induced death or senescence [56]. Finally, membrane alanyl aminopeptidase (ANPEP), a membrane enzyme involved in peptide hydrolysis and glutathione metabolism, was downregulated in our MPR model and highly expressed in the descending thick limb of the loop of Henle. Notably, ANPEP was associated with modulating glutathione redox homeostasis in renal tissue [57]. Another integrative study, associated with the renin-angiotensin-aldosterone system, shows a causal relationship between ANPEP and acute kidney injury, which deserves further clinical investigation [58]. ANPEP has also been associated with diabetic retinopathy and nephropathy in patients with type 2 diabetes mellitus [59]. These results highlight the impact of MPR on renal mitochondria and nephron-specific sites, outlining mitochondria as a potential organelle to be investigated in the context of the developmental origins of renal disorders.

Particularly concerning amino acid metabolism and β-oxidation, our results revealed downregulation of aldehyde dehydrogenase 6 family member A1 (ALDH6A1), which is highly expressed in the proximal convoluted tubule. ALDH6A1 participates in valine and pyrimidine catabolism and is reported as a potential target in renal tumorigenesis [60, 61], also regulating cell proliferation under homeostatic and pathological conditions [62]. Likewise, medium-chain acyl-CoA dehydrogenase (ACADM) initiates mitochondrial fatty acid β-oxidation and was downregulated in MPR kidneys, with predominant expression in the thick descending limb of the loop of Henle. Rare deleterious ACADM variants have been linked to CKD–related metabolic disturbances [63], and mitochondrial dysregulation involving ACADM and IDH2 has been identified in the thick ascending limb of salt-sensitive Dahl rats [64]. Hydroxyacyl-CoA dehydrogenase (HADH), a key β-oxidation enzyme, was downregulated in MPR animals, despite its typical enrichment in proximal and distal tubules, and is associated with inflammation and renal tumorigenesis [65]. Collectively, these findings suggest that MPR impairs mitochondrial amino acid and fatty acid oxidation, disrupting nephron energy metabolism and compromising biosynthetic and maintenance processes in metabolically active tubular segments.

Another key group affected by MPR involves tubulins, structural components of microtubules that form the cytoskeleton. In MPR-exposed kidneys, TUBB2B, TUBA1A, and TUBB5 were upregulated - TUBA1A and TUBB5 predominantly in Bowman’s capsule, and TUBB2B in the terminal collecting duct. Jeruschke et al. (2015) showed that mTOR inhibition affects podocyte cytoskeleton organization via microtubule regulation involving TUBB2B and DCDC2 [66]; Zhang et al. (2025) identified TUBA1A as a mitochondrial dysfunction–related gene in diabetic kidney disease [67]; and Manissorn et al. (2016) demonstrated that TUBA1A protects against cell death and CaOx crystal adhesion in kidney stone disease, while promoting cell proliferation and repair [68]. These findings suggest that MPR-induced tubulin upregulation reflects cytoskeletal remodeling and compensatory repair to metabolic and mitochondrial stress. Consistently, multiomics data in renal ischemia–reperfusion injury identified cytoskeletal-associated markers (e.g., TUBA1A) linked to maladaptive repair and poor outcomes [69]. These findings suggest impaired vesicular transport, particularly in the Bowman’s capsule and collecting duct, potentially disrupting osmolarity regulation and glomerular filtration, as well as the trafficking of hormone-dependent channels (e.g., aquaporins in response to ADH and ion channels regulated by aldosterone). Such alterations may represent early, nephron-specific biomarkers of MPR-induced dysfunction and increased risk of hypertension later in life.

GPX1, which reduces hydroperoxides and H₂O₂, was upregulated in MPR kidneys with high expression in the proximal tubule. This enzyme emerges as a key biomarker of renal programming, given its link to redox balance and pathways associated with increased blood pressure [70]. Epigenetically, these findings suggest an initial adaptive response that may be epigenetically reprogrammed over time, increasing hypertension risk. Notably, GPX1 also shows differential expression in cancers and serves as a prognostic biomarker, including in renal tumors [71]. Regarding Cytochrome C, Somatic (CYCS), a key mitochondrial electron transport chain component, was downregulated overall, with higher expression in the distal tubule and collecting duct. Interestingly, only two studies have associated CYCS with kidney function: mice following oral feeding with Realgar [72] and boric acid administration is effective on severe liver and kidney damage caused by LPS [73], which can be considered a new biomarker of renal DOHaD programming. Finally, ATP1B1 and ATP1A2 (Na⁺/K⁺-ATPase subunits) were downregulated, indicating impaired ion transport and membrane function. ATP1B1 showed high expression along the loop of Henle, distal tubule, and collecting duct, while ATP1A2 was enriched at the beginning of the collecting duct. ATP1B1 can be regulated by nutrition and influence renal ion transport [74]. Through the control of miR-192-5p, ATP1B1 is a functional target that mediates protective effects against renal hypertension [75]. These findings indicate that MPR disrupts electron transport, antioxidant defense, and ion transport, suggesting impaired neuroendocrine regulation and renal development, and highlighting nephron-specific biomarkers for further investigation.

One limitation of this study is the absence of sex-specific analyses. Nevertheless, the findings uncover key biological features - and even potential single-cell mechanisms and biomarkers - associated with early renal programming under maternal malnutrition. These insights establish mechanistic pathways for future studies addressing female responses and aging, offering a strong basis for translational research. Another limitation is the lack of gene or protein expression validation; however, proteomic analysis provides higher sensitivity and reliability, enabling the identification of broader mechanisms that traditional validation methods may overlook. In addition, although the single-cell analysis can provide several insights about the nephron-specific deregulation, our results were conducted based on a mouse dataset, which has a high degree of biological and genomic conservation with rats; however, it is not a rat dataset. Finally, although this study highlights early-life renal programming without assessing long-term consequences, it lays a solid foundation for future investigations into whether maternal protein restriction contributes to chronic kidney disease later in life.

## Conclusion

Therefore, this study identifies both upregulated and downregulated pathways in the kidneys of post-weaning rats exposed to MPR, revealing dysregulated targets potentially linked to renal epigenetic-metabolic programming and an increased risk of CKD. It provides comprehensive insights into the biological, cellular, biochemical, molecular, epigenetic, and nephron-specific impacts of MPR on renal function. We highlight 15 potential biomarkers associated with epigenetic programming and long-term kidney disease risk, offering a valuable foundation for future research on aging-related effects. Interestingly, our results highlight nephron-specific effects in early life in response to MPR, primarily emphasizing impacts on the proximal convoluted tubules and molecule transport, as well as areas of intrinsic regulation (renal corpuscle) and hormone-dependent regulation (distal tubule and collecting duct) associated with microtubule function, metabolism, and stress response. Moreover, this study underscores the critical importance of maternal health during pregnancy and lactation, emphasizing how maternal nutrition shapes offspring health. These findings support future translational and epidemiological studies and may inform public policies aimed at mitigating hunger and food insecurity.

## Supplementary Information

Below is the link to the electronic supplementary material.

Supplementary Material 1 (DOCX 1.92 MB)

Supplementary Material 2 (XLSX 20.7 KB)

Supplementary Material 3 (XLSX 19.6 KB)

Supplementary Material 4 (XLSX 15.3 KB)

Supplementary Material 5 (XLSX 14.0 KB)

Supplementary Material 6 (XLSX 72.1 KB)

Supplementary Material 7 (XLSX 25.2 KB)

Supplementary Material 8 (XLSX 136 KB)

Supplementary Material 9 (XLSX 60.1 KB)

Supplementary Material 10 (XLSX 12.9 KB)

Supplementary Material 11 (XLSX 12.3 KB)

Supplementary Material 12 (XLSX 99.7 KB)

Supplementary Material 13 (CSV 519 KB)

## Data Availability

No datasets were generated or analysed during the current study.

## Declarations

### Generative a.i. and ai-assisted technologies in the writing process

All the text of the manuscript was initially written without Generative AI. Generative AI (ChatGPT) was partially used to correct some English formulations or to improve the formulation of a few sentences.
