# Supplementary material for: Renal proteomics of male offspring exposed to maternal protein restriction: molecular, epigenetic, and nephron-specific signatures of metabolic programming
Source: J Physiol Biochem. 2026 May 7;82(1):48. doi: 10.1007/s13105-026-01189-9 (PMC13149610; doi:10.1007/s13105-026-01189-9)
Supplement: Supplementary file 1 — Supplementary Material 1 (DOCX 1.92 MB) [file 13105_2026_1189_MOESM1_ESM.docx]

**Supplementary Material**

**Renal Proteomics of Male Offspring Exposed to Maternal Protein Restriction: Molecular, Epigenetic, and Nephron-Specific Signatures of Metabolic Programming**

**Danielle Amanda Niz Alvarez¹^a^; Isabelle Tenori Ribeiro¹^a^; Matheus Naia Fioretto¹^a^**; Marina Pereira Pires¹; Luisa Annibal Barata¹; Flávia Alessandra Maciel¹; Luiz Marcos Frediane Portela¹; Renato Mattos¹; Hecttor Sebastian Baptista¹; Pedro Menchini Vitali¹; Vinicius Alexandre Andrade Felipe¹; Marcel Rodrigues Ferreira²; Elena Zambrano³^4^; Patrícia Aline Boer^5^; Luis Antonio Justulin¹

**Supplementary Table 1.** Nutritional information about diets

| **Ingredients** | **Normoprotein**  **(17% protein)** | **Low-Protein**  **(6% protein)** |
| --- | --- | --- |
| **Starch** | 397 | 480 |
| **Casein (84% protein)** | 202 | 71.5 |
| **Dextrin** | 130.5 | 159 |
| **Sucrose** | 100 | 121 |
| **Soy oil** | 70 | 70 |
| **Fiber of pH 101 or pH 102 (microcellulose)** | 50 | 50 |
| **Mixture of salts AIN93Gº** | 35 | 35 |
| **Mixture of vitamins AIN93Gº** | 10 | 10 |
| **L-cystein** | 3 | 1 |
| **Choline bitartrate (41.1% choline)** | 2.5 | 2.5 |
| **Energy Intake (Kcal/g-1)** | 3.96 | 3.96 |

The low-protein diet was prepared by Pragsoluções (PragSoluções, São Paulo, Brazil). All formulations were supplemented with L-cystine as the sulfur-containing amino acid. Vitamin and mineral mixes were adjusted to meet the American Institute of Nutrition (AIN-93G) recommendations for rodent diets. To maintain phosphorus at the same level as in the control casein diet (3 g/kg), monobasic potassium phosphate was added to the mineral mix, ensuring a consistent calcium-to-phosphorus ratio of 1.3 in both diets.


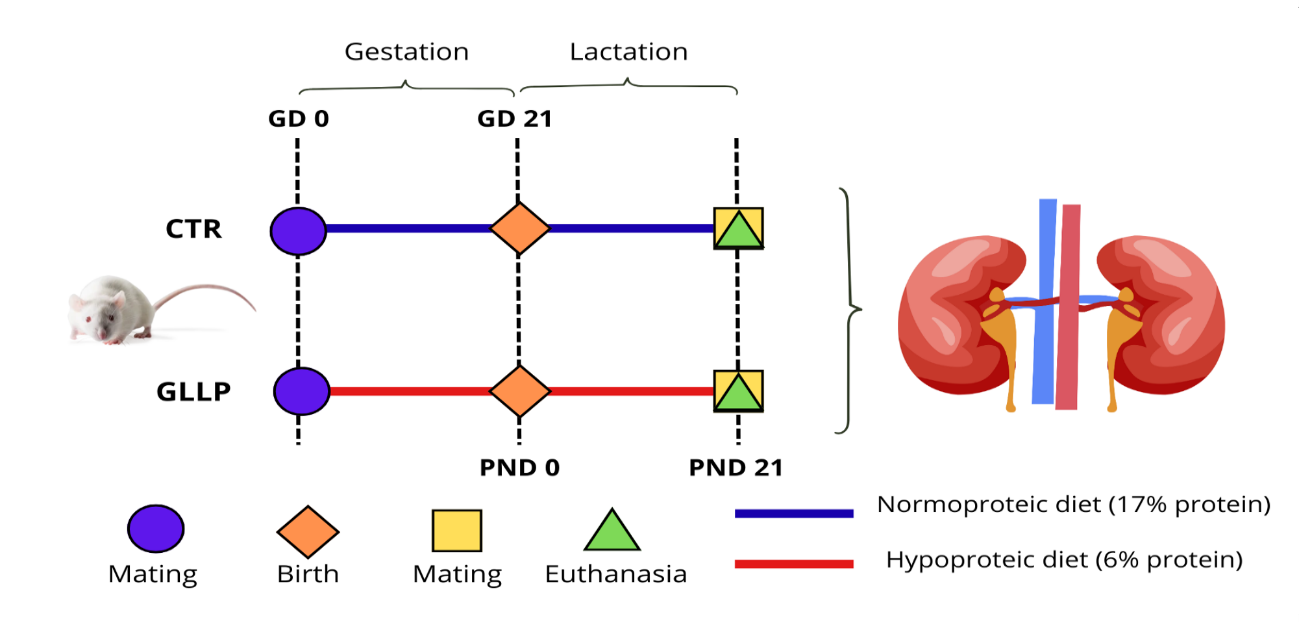


**Supplementary Figure 1.** Experimental design. Male Sprague-Dawley rats were divided into two groups: CTR: rats born to mothers who consumed a normoproteic diet (17% protein) during gestation and lactation; GLLP: rats born to mothers who consumed hypoproteic diet (6% protein) during gestation and lactation. The rats were euthanized at postnatal day (PND) 21.


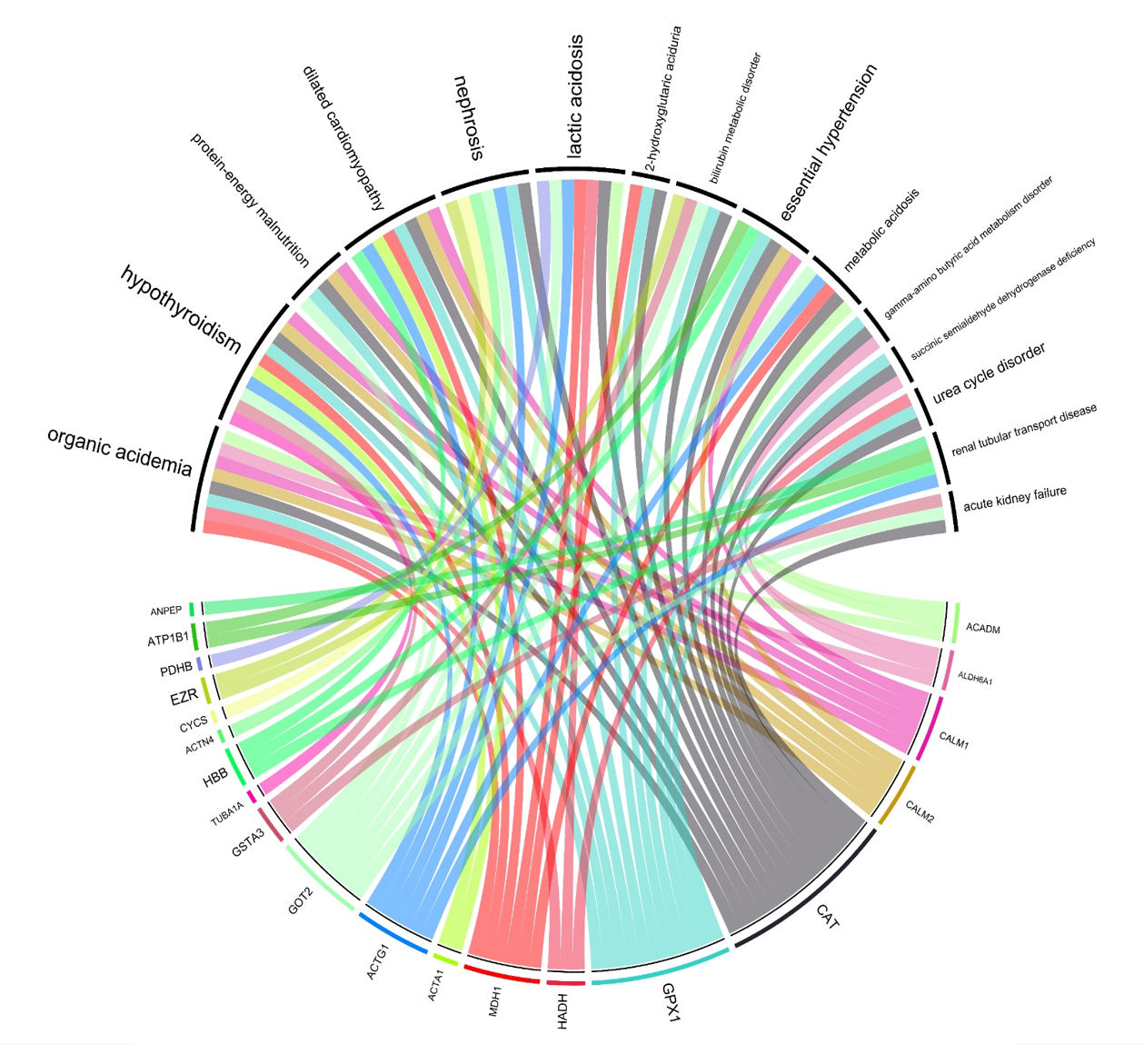


**Supplementary Figure 2.** Interaction network between differentially expressed proteins (DEP) and potential associated diseases. Integration of metabolic/kidney disease-related signatures among proteins identified based on epigenetic potential. Circos graph illustrating the network of associations between identified proteins (below) and diseases (above). Colored connections represent known or predicted links extracted from the same integrated databases, revealing multi-disease associations. Analyses were conducted in R and CircLize. Statistical significance was assessed using the Benjamini-Hochberg method to correct for multiple testing, with a significance threshold of p < 0.05, based on the binary distance of the dendrograms.
